# Best hyperspectral indices for assessing leaf chlorophyll content in a degraded temperate vegetation

**DOI:** 10.1002/ece3.4229

**Published:** 2018-06-25

**Authors:** Yu Peng, Min Fan, Qinghui Wang, Wenjuan Lan, Yating Long

**Affiliations:** ^1^ College of Life & Environmental Sciences Minzu University of China Beijing China

**Keywords:** correlation analysis, degraded vegetation, hyperspectral indices, leaf chlorophyll content

## Abstract

Extensive studies have focused on assessing leaf chlorophyll content through spectral indices; however, the accuracy is weakened by limited wavebands and coarse resolution. With hundreds of wavebands, hyperspectral data can substantially capture the essential absorption features of leaf chlorophyll; however, few such studies have been conducted on same species in various degraded vegetations. In this investigation, complete combinations of either original reflectance or first‐order derivative spectra we conducted a complete combination on either original reflectance or its first‐order derivative value from 350 to 1000 nm to quantify leaf total chlorophyll (Chll), chlorophyll‐a (Chla), and chlorophyll‐b (Chlb) contents. This was performed using three hyperspectral datasets collected in situ from lightly, moderately, and severely degraded vegetations in temperate Helin County, China. Suitable combinations were selected by comparing the numbers of significant correlation coefficients with leaf Chll, Chla, and Chlb contents. The combinations of reflectance difference (*D*_ij_), normalized differences (ND), first‐order derivative (FD), and first‐order derivative difference (FD(*D*)) were found to be the most effective. These sensitive band‐based combinations were further optimized by means of a stepwise linear regression analysis and were compared with 43 empirical spectral indices, frequently used in the literature. These sensitive band‐based combinations on hyperspectral data proved to be the most effective indices for quantifying leaf chlorophyll content (*R* ^2^ > 0.7, *p* < 0.01), demonstrating great potential for the use of hyperspectral data in monitoring degraded vegetation at a fine scale.

## 1 INTRODUCTION

Currently, one‐third of earth's continents are covered by degraded land, with various intensities of vegetation degradation (FAO, 2014). The monitoring of degraded vegetation is an important issue for grazing management, the identification of conservation area, ecological restoration of degraded land across the globe. This requires an accurate and fast estimation of plant physiological parameters at multiple scales (Yuan et al., 2011; Xia et al., 2014). Leaf chlorophyll content (Chll), defined as total chlorophyll content [chlorophyll a (Chla) +chlorophyll b (Chlb)], is an important variable for global plant physiological status monitoring (Malenovský et al., 2013; Houborg, Fisher, & Skidmore, 2015a; Beck et al., 2016). Plant chlorophyll content is one of valuable diagnostic indicators for the early identification and assessment of the overall health of the vegetation, indicating its degradation status (Gottardini et al., 2014; Peng et al., 2014). This will allow us to conduct restoration and revegetation actions where they are required. Remote sensing, including hyperspectral remote sensing, is one of the most common pathways for fast and nondestructive Chll content estimation at leaf and canopy scales (Elarab, Ticlavilca, Torres‐Rua, Maslova, & McKee, 2015; Houborg et al., 2015a). Numerous spectral indices have been developed to estimate leaf Chll and its composition. Hyperspectral data, with its hundreds of wavebands and 1–3 nm resolution, can greatly improve prediction accuracy, have attracted extensive attention and been regarded as a powerful proxy to extract the information of plant physiological parameters. Since then, extensive studies have been conducted aiming at to develop better hyperspectral indices than before (Lu & Lu, 2015; Liang et al., 2016). With the appearances of hyperspectral satellite (Marshall & Thenkabail, 2015), hyperspectral data demonstrate great potential for ecological application. To date, most published hyperspectral indices for estimating chlorophyll content generally use the wavelength domain ranging from 400 to 860 nm, on either original reflectance or derivative value‐based indices (Peng, Gitelson, Keydan, Rundquist, & Moses, 2011). Most spectral indices are only applicable to vegetation types which are developed, subject to site‐specific problems. Numerous indices were developed based on purely statistical analysis. Specific wavelengths selected through this method could change from one location to another, as lack in the consideration of leaf Chll absorption characteristics. It can reasonable to deduce that hyperspectral indices developed on narrow bands sensitive to leaf Chll content could perform better than empirical spectral indices solely based on several bands.

Currently, the first derivative value (FD) is often used to decompose a mixed spectrum and reduce the noise in hyperspectral data (Yao et al., 2015). Many studies have demonstrated the potential of derivative spectra for estimating chemical contents of noncrop vegetation types (Chen, Li, Wang, Peng, & Chen, 2011; Cao, Wang, & Zheng, 2015). Derivative spectral indices are found very sensitive to Chll, among them the first‐order derivative spectra are the best predictors for Chll content (Liang et al., 2016). However, few studies have examined the performance of first‐order derivative spectra or its combinations in estimating leaf Chll content through wavelengths from 400 to 1000 nm, which is the frequently used domain in spectra for most spectral sensors worldwide.

Considering above background analysis, this study uses the entire reflectance data ranges from 350 to 1000 nm, and complete combination of reflectance or its FD, followed by correlation and stepwise regression, which were not used before, to improve hyperspectral indices. The main aim of this study was therefore oriented toward developing suitable hyperspectral indices for estimating leaf chlorophyll content in temperate degraded vegetation. To achieve this key objective, this study will identify the narrow wavebands sensitive to these elements, through the comparison of correlation coefficients among a complete combination of reflectance and its FD. This will be performed across the entire available wavebands. We use a stepwise linear regression analysis for combination optimization. The newly developed indices are then compared with published empirical indices in order to select the best performing hyperspectral indices for the estimation of leaf Chla, Chlb and Chll status. An extensive dataset of in situ hyperspectra and leaf Chla, Chlb, and Chll contents was collected over three degraded intensity vegetation sites in Helin County, Inner Mongolia over a 2‐year period (2012–2013), and also used for simulation and validation.

## 2 MATERIALS AND METHODS

### 2.1 Study area

**Figure 1:**
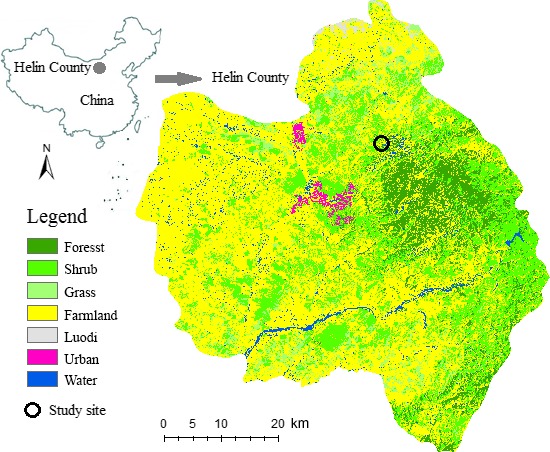
The land use map of Helin County, China, indicates the location of present study

The study was conducted in Helin County, Inner Mongolia, China. Helin County locates at the northern agro‐pastoral ecotone, characterized by a collection of flat plains, hills, and mountains with relatively equal area (Figure 1). The highest elevation was 2031 meters and was a total area of 3401 square kilometers. Helin County has a temperate climate with obvious wet (summer) and dry (winter) seasons. Its annual average temperature is 5.6°C, with a seasonal average temperature of ‐12.8°C in January and 22.1°C in July. The average annual precipitation is 417 millimeters, with approximately 30 millimeters in January and 103 millimeters in July. The average wind speeds are slightly higher in spring and winter than in the summer and fall seasons. The average relative humidity for the whole year does not show obvious seasonal changes. The semi‐arid climate supports sandy vegetations, in which grass and shrubs are predominant in this area.

### 2.2 In‐situ hyperspectral and chlorophyll measurements

In‐situ datasets were collected from degraded sparse‐forest grassland in Helin County (Fig. 1). The degradation intensity of vegetation was classified into three intensities: light, moderate and severe degradation, according to canopy coverage, plant diversity and soil conditions (Peng et al., 2014). Light degraded vegetation has highest canopy coverage (76%), species diversity (richness is 32 and Shannon–Weaner index is 2.36) and soil moisture (Relative weight in 20 cm soil depth, 24%), followed by moderate (52%, 28 and 1.34, 16%) and severe degraded vegetation (33%, 22 and 0.88, 7%). The field measurements were collected on July 2012 and August 2013 at the climax of the growing period in the area of study and under clear sky conditions between 10:00 and 14:00 local time (Beijing Time). Hyperspectral data, from the middle leaf samples of seven plants in each six dominant plant species in light, moderate and severe degradation vegetation, respectively, were recorded using a Hand–Held ASD portable FieldSpec 2 spectrometer (Analytical Spectral Devices Inc., USA). The spectrometer has a spectral range extending from 325 to 1075 nm, and a 1 nm bandwidth. Leaf reflectance values were acquired through a leaf clip attached to the device with an optical fiber. The source of light was integrated in the leaf clip and the black reference panel on the opposing side was used to calibrate the instrument for the reflectance values. Leaf chlorophyll content measurements were punched after leaf reflectance measurements and then analyzed in the field using dual‐beam scanning ultraviolet‐visible spectrophotometers (Ultrospec 3300 pro, Amersham Biosciences, Piscataway, NJ, USA). Arnon's method (1949) was applied to calculate leaf Chla, Chlb and Chll contents after absorption. Totally, there were 126 samples acquired (7 plants multiple by 6 species multiple by 3 intensities) as original reflectances and leaf Chla, Chlb, Chll measurements, respectively. Of these, 64‐pair samples were used for spectral models construction, 80‐pair samples (for empirical indices) and 30‐pair samples (for new indices) were used for validation of every leaf chlorophyll content, respectively.

### 2.3 Hyperspectral indices development and validation

In the raw data, the marginal ranges 325–350 nm and 1,000–1,075 nm from each spectrum were removed due to noise effects. The aim of spectral indices is to construct a mathematical combination of spectral band values for enhancing the information content in regard to the parameter under study. Most published indices (Stagakis, Markos, Sykioti, & Kyparissis, 2010; Inoue, Sakaiya, Zhu, & Takahashi, 2008) are expressed as reflectance (*R*_*i*_) or a first‐order derivative (FD) at a given wavelength, wavelength difference (*D*_ij_), ratio (RR), normalized difference (ND) or inverse reflectance differences (ID). Thus, ten common types of indices based on both original reflectance and derivative spectra, as follows, were used in this study:

(1) $$R=R_{i}$$

(2) $$D_{\mathrm{ij}}=R_{j}-R_{i}$$

(3) $$\text{RR}=R_{j}/R_{i}$$

(4) $$\text{ND}=\left(R_{j}-R_{i}\right)/\left(R_{j}+R_{i}\right)$$

(5) $$\text{ID}=1/R_{j}-1/R_{i}$$

(6) $$\text{FD}=\text{FD}\left(R_{i}\right)$$

(7) $$\text{FD(D)}=\text{FD}\left(D_{\mathrm{ij}}\right)=\mathrm{FD}\left(R_{j}\right)-\text{FD}\left(R_{i}\right)$$

(8) $$\text{FD}\left(\text{RR}\right)=\text{FD}\left(R_{j}\right)/\text{FD}\left(R_{i}\right)$$

(9) $$\text{FD}\left(\text{ND}\right)=\left(\text{FD}\left(R_{j}\right)-\text{FD}\left(R_{i}\right)\right)/\left(\text{FD}\left(R_{j}\right)+\text{FD}\left(R_{i}\right)\right)$$

(10) $$\text{FD}\left(\text{ID}\right)=1/\text{FD}\left(R_{j}\right)-1/\text{FD}\left(R_{i}\right)$$

where *R* is reflectance, FD is first‐order derivative spectra and the suffixes (*i* or *j*) are wavelength(nm). In the entire wavelength domain ranging from 350 to 1,000 nm, these indices were evaluated by correlation analysis with leaf Chll and its composition. The optimum wavelength representing Chll, Chla and Chlb content was identified based on the highest *R* ^2^ between the in‐situ hyperspectral data and leaf Chll contents.

The sensitive bands will be further filtered through stepwise multiple linear regression analysis. Stepwise multiple linear regression analysis can reduce the redundancy collinear spectral variables to a few noncorrelated latent variables, thereby avoiding the potential overfitting problems that are typically suffered with correlation analysis (Yu et al., 2013; Luo et al., 2017).

Still, in order to evaluate our developed hyperspectral indices, we have derived 43 empirical frequently used indices from the published literature. We compared the performance of the empirical indices with newly developed hyperspectral indices by comparing the *R* ^2^ value and its significant level.

The final spectral indices, which were extracted from narrow sensitive narrow bands, passed the statistical significant significance test, performed better than empirical indices, would and can be regarded as a global index which will sufficiently represent leaf Chll content.

## 3 RESULTS

### 3.1 Hyperspectral curves

**Figure 2:**
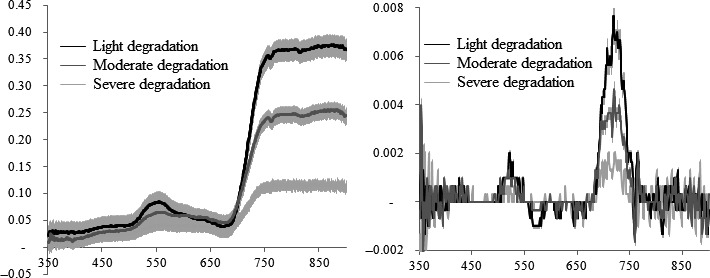
Mean leaf reflectance (left), the first deviation (right) and 95% confidence intervals (in light gray) for the samples collected from the light, moderate and severe degradation vegetations in Helin County, Inner Mongolia, China

We first investigated the hyperspectral curves of degraded vegetations with various degradation intensities and estimated to what degree the spectral response differentiates. It is obviously the reflectances differed along degradation intensities (Figure 2). We performed a *t*‐test for the bands at 350 and 1,000 nm which represent the optical and near‐infrared zones. For each pair datasets, the *p* value was less than 0.05, indicating that the discrepancies between degradation intensities are statistically important. Thereafter, the development of new hyperspectral indices and utilization of empirical spectral indices should be conducted on three degradation intensities. Finally, the indices which have the high consistency and high accuracy across three degradation intensities were selected as the best indices to predict leaf chlorophyll content.

### 3.2 Correlation curves of the combines with Chla, Chlb and Chll

**Figure 3:**
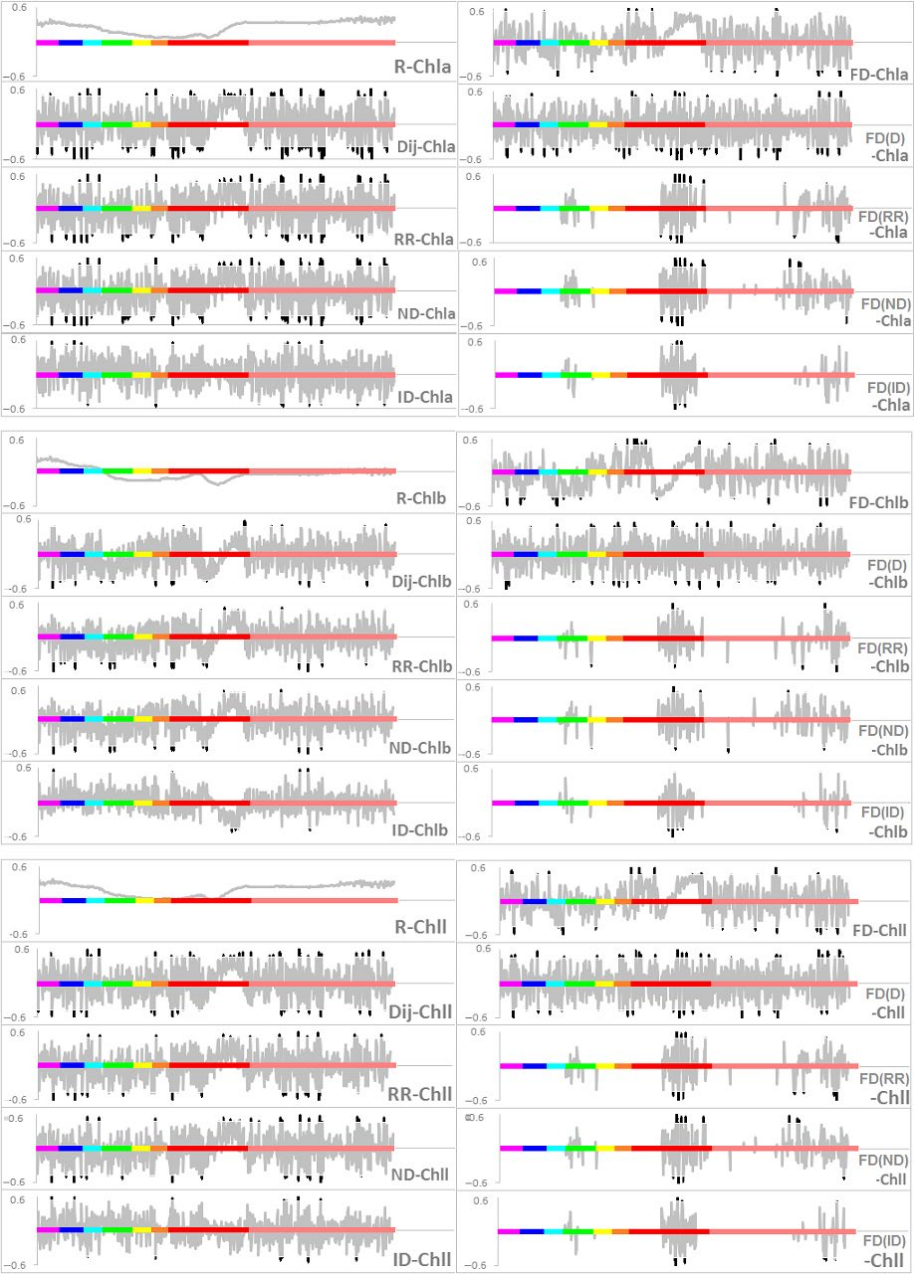
The correlation coefficient curves of combinations of *R*_*i*_, *R*_*j*_ with leaf Chla, Chlb and Chll content, respectively. The x‐axes indicate the wavelength ranges from violet, blue, cyan, green, yellow, orange, red to near‐infrared light (350–1000 nm). The y‐axes indicate the correlation coefficient. The curves in light dark indicate nonsignificant coefficients, in deep dark indicate significant coefficients with *p* < 0.05

Figure 3 presents an indicative subset of the results of the *R*,* D*_ij_, RR, ND, ID, and FD, FD(D), FD(RR), FD(ND) and FD(ID) related with leaf Chla, Chlb and Chll content, respectively. These graphs show the various combinations of reflectance with against plant Chll content, and the presence of correlations. According to Inoue et al. (2008), such plots can provide a significant source of information to correlate the physiological parameters under study. This allows us to optimize the selection of effective wavelengths and bandwidths. The figures indicate that the correlation curves of *R*_ij_ with Chla, Chlb, and Chll are very similar, which can be attributed to the fact that both leaf chlorophyll ingredients are proportionally composed and thus spectroradiometers’ values are proportionally recorded. For correlation curves of FD, its combinations were also similar. Both FD curves and its combinations curves demonstrate a high variability across wavelengths from violet to near‐infrared light.

### 3.3 Development of new hyperspectral indices

By Figure 3, it is obvious the significant coefficients lie in the bands of violet, blue, most red and near‐infrared light. Middle range of red bands is relatively low in the number of significant coefficients, as it is the chlorophyll absorption region. Cyan, green and yellow bands have the relatively few numbers of significant coefficients than other bands. By comparing of the number of significant correlation coefficients with each leaf chlorophyll content, the combinations of *D*_ij_, FD(D), RR and ND for Chla, RR, ND, FD(R) and FD(D) for Chlb, *D*_ij_, RR, ND, FD(R) and FD(D) for Chll, have the most number of significant correlation coefficients and thus were selected as the sensitive combinations to indicate leaf Chla, Chlb and Chll content, respectively.

However, the correlation coefficients of the combinations as *D*_ij_, ND, RR, and FD(*D*) and FD(*R*), vary across the wavelengths. For example, the correlation coefficient for *D*_ij_ with leaf Chlb content is significant in one band yet insignificant in the neighboring regions of the spectrum. Some bands have significant coefficients among several combinations, that is, it is not only significant in the *D*_ij_ curve, but also in the ND and FD(*D*) curves. Thus, the bands with the largest number of significant coefficients among all of the combination curves were identified as the most sensitive bands. The identification of sensitive bands for each combination is also important as it can narrow the wavelength range, and hence may yield a more powerful indicator of plant leaf Chll content. By counting the number of significant correlation coefficients, the sensitive bands for each selected combination were identified. Using this method, a total of 28 sensitive bands were identified for leaf Chll content. We used a stepwise linear regression analysis to further identify the best bands among the 28 sensitive bands selected above. This allowed us to find the best combination of bands for each index. Once this step had been completed, four regression equations were established for leaf Chll, Chla, and Chlb content, respectively (Table 1).

**Table 1:** The stepwise linear regression equations indicate leaf chlorophyll parameter, based on sensitive bands selected from reflectance and FD combinations

Regression equations	*R* ^2^	Adjusted *R* ^2^
Chla = 0.090 − 185.914D_456_ + 89.698D_827_ − 39.711D_811_	0.830	0.808**
Chlb = 0.095 − 32.502D_416_ + 18.779D_760_ − 8.401D_907_	0.769	0.719**
Chll = 0.202 − 217.788D_456_ + 121.746D_827_ − 115.428D_429_	0.839	0.804**
Chla = 0.156 − 4.209ND_900_ − 13.995ND_456_ + 33.639ND_827_	0.790	0.745**
Chlb = 0.06 − 1.285ND_416_ − 5.95ND_630_ − 8.584ND_633_	0.705	0.641**
Chll = 0.251 − 17.704ND_456_ − 4.824ND_416_ + 36.932ND_827_	0.795	0.751**
Chla = 0.202 + 325.288FD_739_ − 130.458FD_924_ − 95.617FD_430_	0.812	0.771**
Chlb = 0.095 − 94.253FD_481_ + 62.661FD_484_ + 40.558FD_679_	0.769	0.720**
Chll = 0.31 + 518.714FD_739_ − 210.946FD_610_ + 116.305FD_928_	0.778	0.730**
Chla = 0.065 − 144.602FDD_428_ + 216.371FDD_498_ + 159.810FDD_489_	0.874	0.847**
Chlb = 0.046 − 37.145FDD_428_ − 82.514FDD_480_ − 33.461FDD_821_	0.876	0.849**
Chll = 0.198 − 155.812FDD_428_ + 471.014FDD_739_ + 177.699FDD_647_	0.870	0.842**

Note. *D* = *D*_ij_, FDD = FD(*D*) = FD(*D*_ij_); D_123_ indicates the *D* value at 123 band; significant level is indicated by *(at 0.05 level) or **(at 0.01 level).

### 3.4 Empirical hyperspectral indices assessment

There are 43 frequently used empirical indices cited in the previous studies. These have been selected as the reference indices. Table 2 lists the correlation coefficients of the 43 empirical indices along with our in‐situ spectral measurements for the three degradation intensities. An obvious fluctuation in correlation coefficients among the different degradation intensities is observed. For Viopt, FD525‐570, MSS‐DVI, RES, SDb and SDr, their values are significantly negative when the degradation intensity is light. However, they become significantly positive when the degradation intensity is severe. Contrary to these indices, NVI and SDy have significantly positive when the degradation intensity is light and significantly negative when intensity is severe. A desirable hyperspectral index, which can be widely and easily used, should perform steadily and accurately under all of the different degradation intensities for the same type of vegetation. Based on this criterion, we considered the consistency and the number of significant correlation coefficients across the three degradation intensities. Three spectral indices were finally selected for leaf Chll estimation ((SDr − SDy)/(SDr + SDy), SDr/SDy, DVI). These indices have high consistency and good performance (Table 2). Also, the comparison of *R* ^2^ values between the optimized stepwise regression indices (which we derived from the complete combinations (Table 1)) and the empirical indices (Table 2), reveals that the *R* ^2^ values of the proposed stepwise regression indices are significantly higher than the best performing empirical indices.

**Table 2:** The correlation matrix of empirical spectral indices with leaf Chll contents along degradation gradient

Indices	Light degradation			Moderate degradation			Severe degradation		
	Chla	Chlb	Chll	Chla	Chlb	Chll	Chla	Chlb	Chll
NDVI705	−0.065	0.098	−0.016	−0.250*	−0.190	−0.232*	0.122	0.084	0.114
mNDVI705	0.113	0.253*	0.174	−0.210*	−0.156	−0.194*	0.077	0.081	0.079
mSR705	0.032	0.219*	0.100	−0.340**	−0.258**	−0.315**	0.074	0.046	0.068
REP	0.171	0.298**	0.234*	0.042	0.029	0.038	−0.118	−0.129	−0.122
VOG1	−0.010	0.181	0.055	−0.342**	−0.255**	−0.315**	0.086	0.055	0.079
VOG2	−0.035	−0.215	−0.101	0.270**	0.174	0.237*	−0.066	0.001	−0.049
VOG3	−0.026	−0.211	−0.093	0.287**	0.191	0.254**	−0.071	−0.004	−0.054
PRI	0.106	0.247*	0.167	−0.317**	−0.224*	−0.286**	0.264*	0.235*	0.259*
OSAVI	−0.152	−0.061	−0.137	−0.108	−0.086	−0.102	0.173	0.122	0.162
NVI	0.228*	0.283**	0.272*	−0.235*	−0.157	−0.208*	−0.320**	−0.329**	−0.326**
NDCI	−0.166	0.007	−0.125	−0.215*	−0.164	−0.200*	0.051	0.000	0.038
RI1 dB	−0.020	0.170	0.043	−0.358**	−0.269**	−0.331**	0.083	0.064	0.078
MCARI1	−0.344**	−0.108	−0.301**	−0.244*	−0.266**	−0.263**	0.217	0.156	0.203
DVI	−0.350**	−0.108	−0.305**	−0.181	−0.206*	−0.199*	−0.083	−0.026	−0.069
TVIBL	−0.388**	−0.215	−0.372**	−0.226*	−0.260**	−0.250*	0.223*	0.159	0.209
GREEN‐NDVI	−0.157	0.039	−0.107	−0.224*	−0.176	−0.211*	0.044	−0.002	0.032
Viopt	−0.321**	−0.109	−0.284**	−0.257**	−0.256**	−0.266**	0.250*	0.174	0.233*
RVI(810,560)	−0.144	0.071	−0.085	−0.283**	−0.226*	−0.268**	0.061	0.006	0.047
RVI(950,660)	−0.200	−0.069	−0.177	−0.190	−0.148	−0.178	0.184	0.136	0.173
RVI(810,660)	−0.195	−0.084	−0.178	−0.217*	−0.188	−0.212*	0.220*	0.150	0.204
NDVI(573,440)	−0.443**	−0.441**	−0.491**	0.129	0.078	0.111	0.099	0.049	0.087
FD730‐525	−0.159	0.003	−0.121	−0.413**	−0.295**	−0.375**	0.110	0.068	0.100
FD730/525	−0.075	0.004	−0.056	−0.249*	−0.145	−0.210*	0.021	−0.069	−0.002
FD(730‐525)/(730 + 525)	−0.110	−0.006	−0.087	−0.059	−0.027	−0.047	−0.005	0.039	0.007
FD730‐570	−0.235*	−0.084	−0.209	−0.389**	−0.268**	−0.349**	0.182	0.127	0.169
FD730/570	0.121	−0.123	0.046	0.517**	0.403**	0.481**	−0.052	0.001	−0.039
FD(730‐570)/(730 + 570)	0.156	0.094	0.152	0.280**	0.287**	0.294**	0.044	0.031	0.041
FD525‐570	−0.250*	−0.244*	−0.276*	−0.121	−0.059	−0.097	0.220*	0.163	0.207
FD525/570	0.105	0.104	0.127	0.068	0.008	0.042	−0.104	−0.104	−0.105
FD(525‐570)/(525 + 570)	−0.370**	−0.221	−0.370**	0.176	0.118	0.172	0.170	0.184	0.175
MSS‐DVI	−0.378**	−0.256*	−0.378**	−0.416**	−0.348**	−0.400**	0.265*	0.199	0.251*
MSS‐PVI	−0.343**	−0.102	−0.298**	−0.087	−0.113	−0.102	−0.023	−0.066	−0.035
MSS‐SARVI	−0.258*	−0.126	−0.241*	−0.196*	−0.163	−0.188	0.200	0.130	0.184
AVHRR‐GVI	−0.219*	−0.199	−0.236*	−0.116	−0.103	−0.114	0.095	0.106	0.099
SDr‐SDb	−0.240*	−0.148	−0.235*	−0.380**	−0.273**	−0.346**	0.227*	0.175	0.215
RES	−0.293**	−0.162	−0.281*	−0.415**	−0.312**	−0.384**	0.234*	0.184	0.223*
SDb	−0.321**	−0.372**	−0.374**	0.083	0.065	0.078	0.222*	0.165	0.209
SDy	0.313**	0.240*	0.323**	−0.068	−0.108	−0.089	−0.225*	−0.196	−0.219*
SDr	−0.272*	−0.195	−0.275*	−0.354**	−0.254**	−0.322**	0.231*	0.177	0.219*
SDr/SDb	0.078	0.189	0.125	−0.422**	−0.301**	−0.383**	−0.136	−0.019	−0.107
SDr/SDy	−0.261*	−0.208	−0.273*	−0.031	−0.102	−0.065	−0.213	−0.254*	−0.225
(SDr−SDb)/(SDr+SDb)	0.126	0.209	0.169	−0.357**	−0.249*	−0.321**	0.117	0.145	0.126
(SDr‐SDy)/(SDr+SDy)	0.080	0.070	0.085	0.234*	0.218*	0.235*	0.227*	0.199	0.222*

Notes. The bold indices indicate the indices which have consistence and significant correlation coefficients across all gradients.

Significant level is indicated by *(at 0.05 level) or **(at 0.01 level).

In order to conduct an empirical index‐based leaf Chll content assessment, we must establish a suitable equation for each of the three selected indices from Table 2. The linear regression equations were established (Table 3) based on field survey leaf parameters (Chll, Chla, Chlb), and the corresponding empirical indices ((SDr − SDy)/(SDr + SDy), SDr/SDy, DVI) for all three degradation intensities. This indicates that all three empirical indices can predict leaf Chll content at a statistically significant level, except for the DVI for leaf Chlb content.

**Table 3:** The predictions for leaf chlorophyll contents from the empirical spectral indices selected in Table 2

Indices	Chla	*R* ^2^	Chlb	*R* ^2^	Chll	*R* ^2^
(SDr‐SDy)/(SDr+SDy)	y = 0.4585x − 0.3008	0.0546**	y = 0.3312x − 0.2756	0.0475*	y = 0.7897x − 0.5765	0.0553*
SDr/SDy	y = −0.0008x + 0.1393	0.0681*	y = −0.0003x + 0.0497	0.0431*	y = −0.0011x + 0.189	0.0744*
DVI	y = −2E‐05x + 0.2453	0.1222**	y = −4E − 06x + 0.0804	0.0333	y = −2E − 05x + 0.3133	0.093**

Note. Significant level is indicated by *(at 0.05 level) or **(at 0.01 level).

### 3.5 The validation of the selected empirical indices and the newly developed hyperspectral indices

**Figure 4:**
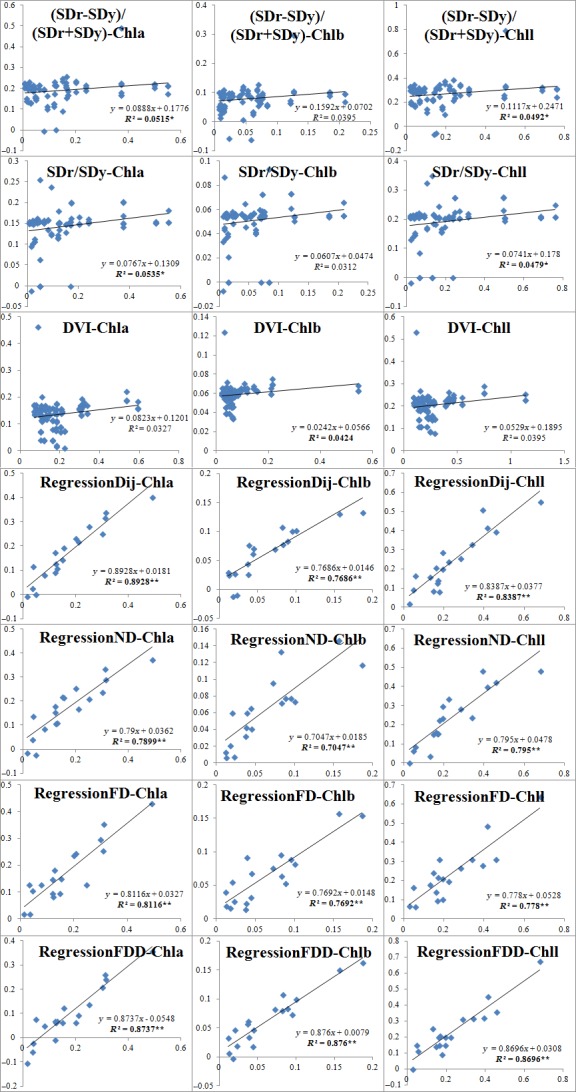
Linear regression for field survey and predicted values of Chla, Chlb, and Chll based on newly developed and empirical spectral indices in various degraded vegetations in Helin County, Inner Mongolia, China. Bold determining coefficients (*R* ^2^) indicate a significant level at 0.05 level (*) or 0.01 level (**)

We used the newly developed (Table 1), and selected empirical (Table 3) spectral indices to predict plant Chll content on other test samples. Based on both the predicted values for each leaf chlorophyll parameter, and those from the field survey, the linear regression and correlation coefficients were established and calculated. In total 21 graphics were created (Figure 4) showing the relation between predicted values and those measured in the field. In most cases, the *R* ^2^ of the empirical model predictions was considerably lower than that calculated from the newly developed models. Even this, the empirical models also statistically satisfy the plant parameter assessment, as indicated by significant correlation coefficients. It is also interesting that the leaf Chla content can be perfected better predicted by both the new and empirical indices, than the other two leaf parameters.

## 4 DISCUSSION

It is convenient to estimate leaf Chll content by means of spectral indices retrieved from observed reflectances by a handheld spectrometer. The complete combinations of reflectance and its first‐order derivative value across the entire band acquired by the ASD spectrometer were constructed. The sensitive bands were identified, and the most suitable models of combined sensitive bands were further selected. The results demonstrate that the newly developed models perform better than solely empirical spectral indices in estimating plant leaf Chla, Chlb, and Chll contents.

Most previous studies developed spectral indices based on visible bands ranging from 400 to 760 nm, on either original reflectance or derivative value‐based indices (Peng et al., 2011). This is because chlorophyll strongly absorbs light at blue (400–500 nm) and red (600–700 nm) regions, and does not include light in the green, orange (500–600 nm), and near‐infrared regions (Houborg et al., 2015b; Beck et al., 2016; Sonobe & Wang, 2017). Wider spectra may capture more information of leaf physiological status. Our study demonstrated several bands beyond 760 nm are also highly sensitive to leaf Chll content. The NIR reflectance, is known to be affected by leaf anatomical structure such as leaf thickness, cell walls, and intracellular air spaces (Slaton, Hunt, & Smith, 2001), could also indicate leaf Chll content (Pastorguzman, Atkinson, Dash, & Riojanieto, 2015), more obviously under the condition of high chlorophyll concentrations (Gitelson & Merzlyak, 2003). Compare to limited combination on original reflectance within visible region in previous some studies, we conducted a complete combination on either original reflectance or its first‐order derivative value, through wavelengths from 350 to 1,000 nm. It has increased the possibility to find more suitable combinations than limited wavelengths used by before.

Many empirical spectral indices are derived from satellite imagery, as 43 indices used in this study, usually encounter the problem of confound signals. The compound signals which satellite sensors received are confounded by the influence of atmospheric effects, vegetation characteristics and background reflectance (Daughtry, Walthall, Kim, & de Colstoun, 2000). The first‐order derivative spectra, which may be calculated approximately by dividing the difference in reflectance between successive wavebands, can eliminate background noise and resolve overlapping spectral features (Aneece, Epstein, & Lerdau, 2017). FD was widely used in various indices for estimation vegetation parameters with a considerable higher accurate (Chen et al., 2011; Cao et al., 2015) than other indices. In the present study, the FD‐based indices were great success in estimating plant leaf Chll and its composition, even we conducted measurements at leaf level which has not background noise and resolve overlapping spectral matters. Thus we strongly suggest FD should be used as an independent variable to estimate plant leaf Chll content.

A high consistence in estimating plant parameters across various degraded vegetations should also be a criterion for desirable spectral indices (Lu & Lu, 2015). We used three intensities of degraded vegetations to test the consistence and credibility of proposed spectral indices. Discrepancies in reflectance and its FD tend to be more pronounced with degradation intensity increase, may partly be explained by the increase in vertical Chll distribution in light degradation vegetation (Gitelson, Peng, Arkebauer, & Schepers, 2014) than severe degraded vegetation. In the severe degraded vegetation, with decreases in canopy Chll content, the absorption capacity also decreases, reflected by lower reflectance values in visible and red edge spectrum than those in the light degraded vegetation (Peng et al., 2014). A desirable hyperspectral index, which can be widely and easily used, should perform steadily and accurately under all of the different degradation intensities for the same type of vegetation. Using degraded vegetations with various degradation intensities, can therefore assess the consistence and accuracy of empirical indices, as significant differences were observed in either reflectance or leaf Chll contents among degradation intensities.

The identification of narrow‐specific bands is also contributed to the perfect results. By correlation curves comparison and stepwise linear regression analysis, the sensitive bands to leaf Chll content have been identified in the present study. For Chla, the sensitive bands are concentrated on 450, 820 and 910 nm. For Chlb, they are 416, 480, 630 nm, for Chll, they are 450, 739 and 827 nm. These sensitive bands are located in the characterized domain of each leaf Chll parameter (Schlemmer et al., 2013; Sanches, Filho, & Kokaly, 2014). It is argued that the narrow bands can capture the Chl‐a reflectance red minimum and near‐infrared peak, estimate Chl‐a concentrations well (Beck et al., 2016). Our study confirmed this deduction. It is valuable to notice that the selection of sensitive bands by stepwise linear regression can greatly improve the predictive performance. As a “full spectrum” method, stepwise linear regression can not only efficiently deal with the strong multi‐collinearity problem, but also considers the covariance problem in the model response/dependent variable(s) (Yu et al., 2013; Luo et al., 2017). Therefore, it is better to deal with potential confounding factors rather than employ a simple index‐based approach.

The present study has considerable applicable potential for practice. Compared with multiple‐spectral imagery, the hyperspectral data gained by handheld portable device has the advantages of high spectral resolution, low labor cost, and less affected by atmosphere layer and background environment. It is more suitable when carrying out repeat measuring on fine‐habitat vegetations over large areas, especially for croplands, grasslands and desert vegetation. With the appearance of more and more satellite‐based hyperspectral data, that is, Hyperion onboard Earth Observing‐1 (EO‐1), with 10‐nm spectral information from 350 to 2,500 nm, and several other satellite‐based hyperspectral sensors, a robustness index for quantifying chlorophyll concentration would have wide application. In addition, the combination of hyperspectral data and satellite/airborne imagery can greatly improve the interpretation precision. This will extend the usage of hyperspectral data to a wider scope, as eco‐restoration, eco‐condition assessment, and precise agriculture.

## 5 CONCLUSION

Complete combinations (value at a given wavelength, wavelength difference, value ratios, normalized differences, and inverse differences) based on either original reflectance or first‐order derivative spectra have been developed to quantify leaf chlorophyll and its composition content using three datasets collected in‐situ from light, moderate and severe degraded vegetations in temperate Inner Mongolia, China. The best combinations identified have further been optimized by sensitive bands selection and stepwise linear regression analysis, and were compared with the 43 empirical indices frequently used in the literature. By validating, the proposed indices proved to be the most effective indices for quantifying chlorophyll contents (*R* ^2^>0.7 and *p* < 0.01), demonstrating great potential for using hyperspectral data in vegetation physiological monitoring at a fine scale. While these, hyperspectral indices are spectrally very narrow and can be applied only when the spectrometer has a very high spectral resolution of 1–3 nm. The new understandings obtained in this study may help to improve the potential of hyperspectral data for world degraded vegetation monitoring. Using these proposals, hyperspectral indices can improve the data quality of satellite/airborne imageries through scale conversion. Future work will encompass more sensors other than hyperspectral devices from satellite, airborne, and LiDAR, to make an application and comparison on plant physiological assessment of desert, grassland, and cropland vegetations. Such research will help us to better understand the dependability of hyperspectral models and to extend the scope of its application.

## AUTHOR CONTRIBUTIONS

Yu Peng designed and performed the study. Min Fan performed the analysis of hyperspectral and field survey data. Qinghui Wang, Wenjuan Lan and Yating Long collected and pre‐processed the hyperspectral and field survey data. All authors discussed the results and contributed to the manuscript.
